# Postural orthostatic tachycardia syndrome in patients of orthostatic intolerance symptoms: an ambispective study

**DOI:** 10.3934/Neuroscience.2021004

**Published:** 2020-12-08

**Authors:** Dinesh Chouksey, Pankaj Rathi, Ajoy Sodani, Rahul Jain, Hashash Singh Ishar

**Affiliations:** Department of Neurology, Sri Aurobindo Medical College and Post Graduate Institute, Indore, M.P. India

**Keywords:** Postural orthostatic tachycardia syndrome, POTS, Head-up tilt table test, HUTT, POTS with syncope, neurocardiogenic syncope

## Abstract

**Background:**

A Postural orthostatic tachycardia syndrome (POTS) is infrequently diagnosed in routine practice because of the variable range of symptoms that could be seen in cardiac rhythm disorders, vertigo, chronic fatigue syndrome and anxiety panic disorder. POTS is a chronic debilitating condition that affects day to day efficient working of an individual. We have planned a study to look for POTS in patients who are having orthostatic intolerance symptoms and underwent a head-up tilt table test (HUTT).

**Aim:**

To study the prevalence of POTS in patients of orthostatic intolerance (OI) symptoms and to analyze symptomatology, its association with neurocardiogenic syncope (NCS), and its outcome.

**Methods:**

We reviewed the medical records of 246 patients presented with symptoms of OI seen at our centre from January 2010 till March 2019. Out of them, 40 patients included, those qualifying the criteria for POTS on HUTT.

**Results:**

The mean age of the cohort was 25.90 ± 10.33 years with a range of 15 to 55 years, and males comprised 52.5% (21/40) of total patients. The most frequent presenting orthostatic symptoms of POTS patients are loss of consciousness (77.5%), lightheadedness (75%), and palpitation (67.5%). A total of 18 patients (45%) had coexisting neurocardiogenic syncope.

**Conclusion:**

POTS is a prevalent condition and have a significant impact on the quality of life, and the majority of patients may not present with OI symptoms during HUTT. We have to keep this possibility in young patients of transient loss of consciousness because it may coexist with NCS.

## 1. Introduction

Orthostatic intolerance (OI) defined as the development of symptoms in standing positions which are relieved when a person adopts a supine posture. Orthostatic intolerance patients frequently complain of dizziness, lightheadedness, palpitations, exercise intolerance, disabling fatigue, headache, blurred vision, anxiety, nausea, cold sweating, chest discomfort, and dyspnea. Syncope and presyncope are distant ends of this spectrum of the disorder [1].

POTS is a clinical syndrome of orthostatic intolerance characterized by a heart rate (HR) increment of 30 beats/min or heart rate (HR) >120 beats/min, within 10 min of standing or on head-up tilt table (HUT), and in the absence of orthostatic hypotension (a decrease in systolic blood pressure (BP) of 20 or more mm Hg and/or decrease in diastolic BP of 10 or more mm Hg) [2]. However, Stewart et al. [3] emphasized that chronic persistent day-to-day OI symptoms are the most notable clinical characteristic of POTS. Likewise, other researchers reported that patients who developed chronic OI symptoms on standing in clinical situations even though they did not have symptoms during the head-up tilt-table test [4],[5].

POTS symptoms are lightheadedness, palpitations, tremor, cognitive impairment, brain fog, and syncope can also occur; non-orthostatic symptoms, such as migraine, fatigue, exercise intolerance, disturbed sleep, visual disturbances, phonophobia and gastrointestinal complaint such as nausea, are also common [6]. Symptoms are frequently aggravated by heat and exercise [7]. Even activities of daily living such as taking a bath or housekeeping work may significantly increase symptoms, resulting in fatigue. This can pose profound limitations on functional capacity [8]. It is imperative to keep this (POTS) entity into consideration because many patients are misdiagnosed as anxiety, panic disorder, depression, and chronic fatigue syndrome [9]. Presyncope is more common than syncope in POTS, but it is not infrequent that POTS coexists with episodes of neurally mediated syncope [10]. The neurally mediated syncopal syndrome includes situational syncope, carotid sinus syndrome, and neurocardiogenic syncope (NCS). NCS is the most frequent cause of syncope in both children and adults, accounting for 50–66% of unexplained syncope [11]. Up to 30% of cases with POTS can have coexistent neurocardiogenic syncope [12]. NCS is caused by an abnormal or increased autonomic response to different stimuli, of which the most common are standing and emotion [11]. Syncope is associated with injuries due to falls or motor vehicle accidents. It poses a potential danger if episodes occur during driving, operating heavy machinery [11]. Head-up tilt-table test (HUTT) with nitroglycerin (NTG) challenge can diagnose NCS in 63% of unexplained recurrent loss of consciousness [13]. Postural orthostatic tachycardia syndrome may be the triggering factor for syncope if they are coexisting [14].

POTS clinical manifestations are varied and nonspecific, probably reflecting various underlying pathophysiological mechanisms [15]. The multiple factors like moderate autonomic dysfunction, increased sympathetic tone, inadequate venous return or excessive blood venous pooling, and severe deconditioning may be responsible for POTS symptoms.

POTS patients often advised for extensive investigation and fragmented management provided by various specialists due to its complexity and heterogeneity in the presentation [16].

The present study describes the clinical presentation, their responses on the head-up tilt table test, and the outcomes of these patients.

## 2. Materials and methods

It is a cross-sectional ambispective study approved by our Institutional ethical and research committee. We reviewed data of 246 patients with orthostatic intolerance seen at our centre from Jan 2010 to March 2019. Compliance with Ethical Standard: Approved by Institute Research and Ethical Committee name SAIMS Indore, SAIMS/IEC/2019/34, Dated 24/04/2019.

### 2.1. Aim

To find out POTS in patients of orthostatic intolerance by doing a head-up tilt table test.

### 2.2. Objectives

1. To study the prevalence, symptoms, and outcomes of POTS patients; 2. To study the POTS patient's orthostatic symptoms during head-up tilt table test; 3. To study the coexistence of POTS with neurocardiogenic syncope.

### 2.3. Inclusion criteria

Patients of orthostatic intolerance diagnosed as postural orthostatic tachycardia syndrome during head up tilt table test were included in the study.

### 2.4. Exclusion criteria

Those who are having abnormal cardiac rhythm, valvular heart disease, carotid stenosis, and ECG evidence of coronary artery disease excluded.

We have reviewed demographic information, presenting symptoms, the tilt-table response from the medical records of these patients. Treatment response was assessed by mobile communication and by direct patient contact where it was feasible.

Postural tachycardia syndrome defined as an increase in heart rate 30 beats per minute within the first 10 minutes of upright tilt, unaccompanied by any decrease in blood pressure with or without symptoms.

NCS defined as a transient loss of consciousness due to cerebral hypoperfusion [17].

The criterion for the diagnosis of NCS included a HUTT response consistent with NCS (a sudden decrease in heart rate and/or drop in blood pressure) that reproduced a patient's symptoms of recurrent transient loss of consciousness with spontaneous recovery [18].

### 2.5. Protocol for HUTT

The HUTT performed as per the Italian protocol [19]. This protocol is divided into an initial drug-free passive phase (20 minutes), and nitroglycerin 400 µg (NTG) spray provocation phase (20 minutes) of passive 70° tilt on a tilt table.

Monitoring: The blood pressure (BP) recorded in the right arm with an automated BP cuff at rest, and after that at every five-minute interval, guided by the patient's symptoms, additional recordings were allowed. The 24 channel Electroencephalography (EEG) recorded without interruption during cool off, initial drug-free passive phase, and NTG provocation phase. EEG using the 10/20 system of electrode placement. The ocular movements recorded on two channels and three channels deployed for the monitoring of EMG (chin and bilateral deltoid). ECG was monitored on a Philips Multipara Monitor and recorded on the EEG system by fixing electrodes on the ventral aspect of the forearm one centimetre proximal to distal wrist crease bilaterally.

End points of the HUTT: 1. Precipitation of clinical symptoms and signs; 2. The occurrence of arrhythmia or symptomatic bradycardia or asystole (lasting for ≥3 seconds) with or without NTG; 3. No symptoms after 20 minutes of post-NTG; 4. Fall in systolic BP of 40 mmHg or more; 5. Patients withdraw consent to continue further testing.

## 3. Statistical analysis

The data analyzed by using SPSS 17.0 version. Descriptive and inferential statistics used results for continuous variables presented as mean ± standard deviation while categorical presented in numbers (%). The age, gender, symptoms, and hemodynamic parameters of POTS patients evaluated. Student's t-test used to investigate the comparison in hemodynamic parameters. The probability value p ≤ 0.05 considered as significant.

## 4. Results

During the study period, 246 patients underwent the tilt-table test, and 40 of them (16.26%) were diagnosed with POTS. Females comprised 47.5% (19/40) of the entire POTS patient group, with a female to male ratio of 0.9:1. The mean age of POTS patients was 25.90 ± 10.33 years with a range of 15 to 55 years. Table 1 summarizes the clinical features and precipitating factors in these patients.

The most frequent presenting orthostatic intolerance symptom of POTS patients were loss of consciousness (77.5%), lightheadedness/dizziness (75%), palpitation (67.5%),weakness (52.5%) and headache (40%). Prolonged standing found to be the most common precipitating factor (42.5%), and migraine (22.5%) was the commonest comorbidity associated with POTS (Table 1).

Table 2 demonstrates the hemodynamic parameters on the tilt test at rest and during the first 10 minutes in the drug-free passive phase of HUTT in POTS patients (n = 40). There was a highly significant increase in heart rate (p ≤ 0.001) during the first 10 minutes in the drug-free passive phase of tilt without substantial change in the systolic and diastolic blood pressure, qualifying for POTS and mean the difference in heart rate was ≈34 beats per minute.

The patients (n = 40) further divided into two groups depending on whether they manifested orthostatic intolerance symptoms during the tilt-table test: the symptom-positive group (n = 4) and the symptom-negative group (n = 36) (Table 3). We compared the results of the tilt-table tests between these groups. There were no significant differences in heart rate (p > 0.05) and blood pressure (p > 0.05) at resting and within the first 10 minutes of tilt in the drug-free phase of HUTT between POTS patients, with and without orthostatic symptoms during the test.

**Table 1.** Baseline clinical characteristics of the study population.

Clinical Characteristics of the Subjects		Responses (N = 40)	
		Frequency	Percent
Age (Mean ± Std. Deviation)		25.90 ± 10.33year	
Age (Median)		22.50 years	
Sex	Male	21	52.5%
	Female	19	47.5%
Symptoms of orthostatic intolerance	Lightheadedness	30	75.0%
	Loss of consciousness	31	77.5%
	Headache	16	40.0%
	Weakness	21	52.5%
	Palpitation	27	67.5%
	Tremulousness	11	27.5%
	Blurring of vision	5	12.5%
	Tingling in all four limbs	1	2.5%
	Shortness of breath	10	25.0%
	Nausea	4	10.0%
	Hyperhidrosis	5	12.5%
Precipitating factor	Micturition	5	12.5%
	Prolonged standing	17	42.5%
Comorbid Condition	Diabetes Mellitus	0	0.0%
	Hypertension	1	2.5%
	Migraine	9	22.5%

**Table 2.** Hemodynamic parameters in tilt test at rest and first 10 minutes of the drug-free passive phase of HUTT.

Parameter	Measuring Position	Scatter Mean ± SD	Mean Diff	t-statistic	p-value (LOS)
Heart Rate	Resting	73.18 ± 13.51	33.97 beat/m	36.32	p < 0.001^#^
	Within the first 10 min of tilt (Passive phase of HUTT)	107.15 ± 15.20			
SBP (mmHg)	Resting	119.38 ± 16.22	0.95 mmHg	0.35	p > 0.05^⨂^
	Within the first 10 min of tilt (Passive phase of HUTT)	118.43 ± 15.45			
DBP (mmHg)	Resting	70.95 ± 11.09	3.80 mmHg	1.67	p > 0.05^⨂^
	Within the first 10 min of tilt (Passive phase of HUTT)	74.75 ± 10.55			

Note: ^#^The mean differences are highly significant at the 0.001 level of significance. ^⨂^The mean difference is not significant at the 0.05 level of significance. The degrees of freedom are 39. (Mean Diff: Mean Difference; min: Minute; SD: Standard Deviation; LOS: Level of Significance; HR: Heart rate; SBP: Systolic blood pressure; DBP: Diastolic blood pressure; HUTT: Head up tilt table).

18 (45%) patients developed clinical features consistent with both POTS and NCS during the NTG provocation phase of HUTT. Table 4 demonstrates the comparison in resting and post NTG blood pressure and heart rate between POTS patients with and without NCS. The analysis showed that there were no statistically significant differences (p ≤ 0.05) in resting minimum systolic and diastolic blood pressure and minimum heart rate between POTS patients with and without NCS. There was a highly significant difference in post-NTG minimum systolic and diastolic blood pressure and minimum heart rate between POTS patients with and without NCS. POTS with NCS patients demonstrated a neuro-cardiogenic pattern on NTG provocation phase of HUTT manifested by sudden fall in heart rate and blood pressure, reproducing symptoms that were similar to the patient's spontaneous episodes. The average time of onset of the neuro-cardiogenic symptom (Mean ± Standard Deviation) during head-up tilt among eighteen cases of POTS with NCS was 25.03 ± 2.87 with a range from 20 to 32 minutes.

**Table 3.** Comparison of heart rate and blood pressure at resting and within first 10 minutes of tilt in the drug-free passive phase of HUTT between POTS patients, with and without orthostatic symptoms.

Measuring Position & Parameter		Symptoms of OI^＄^	Scatter	95.0% CI for Mean		p-value (LOS)
			Mean ± SD	LB	UB	
Resting (supine)	HR beat/m	Absent	73.31 ± 14.11	68.53	78.08	p > 0.05^⨂^
		Present	72.00 ± 6.93	60.98	83.02	
	SBP mmHg	Absent	119.03 ± 15.89	113.65	124.40	p > 0.05^⨂^
		Present	122.50 ± 21.44	88.38	156.62	
	DBP mmHg	Absent	70.78 ± 11.38	66.93	74.63	p > 0.05^⨂^
		Present	72.50 ± 9.15	57.95	87.05	
Within the first 10 minutes of the drug-free passive phase of HUTT	HR beat/m	Absent	106.94 ± 15.79	101.60	112.29	p > 0.05^⨂^
		Present	109.00 ± 9.59	93.74	124.26	
	SBP mmHg	Absent	119.36 ± 14.57	114.43	124.29	p > 0.05^⨂^
		Present	110.00 ± 22.82	73.69	146.31	
	DBP mmHg	Absent	74.83 ± 10.48	71.29	78.38	p > 0.05^⨂^
		Present	74.00 ± 12.83	53.58	94.42	

Note: ^＄^Orthostatic intolerance, ^⨂^The mean difference is not significant at the 0.05 level of significance. The degrees of freedom are 38. (Mean Diff: Mean Difference; HUTT: Head-up tilt table; OI: Orthostatic Intolerance; SD: Standard Deviation; LB: Lower bound; UB: Upper bound; LOS: Level of Significance).

**Table 4.** Comparison in resting and post NTG blood pressure and heart rate between POTS patients with and without NCS.

Measuring Position & Parameter		POTS patients	Scatter	95.0% CI for Mean		p-value (LOS)
			Mean ± SD	LB	UB	
Resting (supine)	HR (beat/min)	Without NCS	74.45 ± 11.87	69.19	79.72	p > 0.05^⨂^
		With NCS	71.61 ± 15.50	63.91	79.32	
	SBP (mmHg)	Without NCS	120.45 ± 17.57	112.67	128.24	p > 0.05^⨂^
		With NCS	118.06 ± 14.80	110.70	125.41	
	DBP (mmHg)	Without NCS	72.41 ± 12.35	66.93	77.89	p > 0.05^⨂^
		With NCS	69.17 ± 9.36	64.51	73.82	
NTG Provocation phase (minimum)	HR (beat/m)	Without NCS	93.82 ± 18.79	85.49	102.15	p < 0.001^#^
		With NCS	59.67 ± 25.91	46.78	72.55	
	SBP (mmHg)	Without NCS	108.32 ± 17.47	100.57	116.06	p < 0.001^#^
		With NCS	78.06 ± 15.36	70.42	85.70	
	DBP (mmHg)	Without NCS	64.41 ± 16.14	57.26	71.56	p < 0.001^#^
		With NCS	42.11 ± 21.47	31.43	52.79	

Note: ^#^The mean differences are highly significant at the 0.001 level of significance. ^⨂^The mean difference is not significant at the 0.05 level of significance. The degrees of freedom are 38. (Mean Diff: Mean Difference; NCS: Neurocardiogenic syncope; SD: Standard Deviation; LOS: Level of Significance; POTS: Postural orthostatic Tachycardia syndrome; HUTT: Head up tilt table; NTG: Nitroglycerin).

The treatment modalities used as per our departmental protocol based on a consensus statement of Heart Rhythm Society [20]. Initial treatment consisted of various non-pharmacological measures like aerobic and resistance training as well as increased dietary fluids and sodium. Pharmacotherapy initiated later on with βeta-blockers, selective serotonin reuptake inhibitors, fludrocortisone [10]. The second and third-line medications like octreotide, erythropoietin, and pyridostigmine not given to any patient. Response to treatment assessed by collecting information about the subjective symptoms such as the ability to remain on feet, degree of improvement, standing time, ability to work in the patient's occupation or at home, ability to withstand orthostatic stresses during standing work and well being on mobile communications and if feasible direct patient inquiry. The treatment considered successful if the patient reported that it provided symptomatic relief and feeling of well being. We were able to contact only sixteen patients because most of the contact numbers changed. Out of these, 15 patients responded to non-pharmacological and first-line treatment with beta-blockers, selective serotonin reuptake inhibitors, and fludrocortisone. One patient had no response to treatment and lost follow up.

## 5. Discussion

The patients of postural tachycardia syndrome have orthostatic symptoms and tachycardia without orthostatic hypotension. The nonspecific clinical symptoms and the absence of orthostatic hypotension have likely resulted in a lack of identification of this syndrome by both clinicians and investigators [21].

The common orthostatic presenting symptoms of POTS in our study were lightheadedness, loss of consciousness, palpitations, weakness, headache, and visual symptoms, which were similar to those reported by Thieben MJ et al. [4]. The prevalence of POTS in our study population is 16.27%, and in another study by Hyung Lee et al., it was 19% [22].

The HR increment of ≈34 bpm during HUTT was consistent with the previous study, which showed an increase of HR by 33.0 bpm [6]. The HR increase a compensatory mechanism to maintain systolic blood pressure and cerebral perfusion at an adequate level, and it is also indicative of excessive sympathetic activation [10]. 42.5% of the total patients experienced prolonged standing as the most familiar precipitating event for OI symptoms. In contrast to our results, 80% of patients experienced prolonged standing as the most familiar precipitating event for OI symptoms as reported by Sandroni P et al. [6]. A frequently reported comorbidity in POTS patients is migraine, as well as other chronic headache types. Intractable migraines often lead to physical inactivity, which may worsen orthostatic intolerance, and conversely, the increased sympathetic activity associated with POTS may add to increased frequency of headaches [10]. We observed migraine as the most common comorbidity in 22.5% of our POTS patients. The results are as per the study done by Thieben MJ et al. [23] and reported in 27.6% of patients. We recommend checking the postural orthostatic tachycardia in patients of nonspecific daily headache, low concentration with generalized fatigue in day-to-day working. In our study males (52.5%) are affected more than females; however, in the other two studies involved males were 37.5% and 49%, that is a contrast to our findings [24],[25].

Most of our POTS patients (n = 36, 90%) manifested no orthostatic intolerance symptoms during the tilt table test. Thieben et al. and Peltier et al. recruited patients who developed chronic day to day orthostatic intolerance symptoms on standing in clinical situations even if they showed no symptoms during the head-up tilt-table test [4],[5]. In another study only, 25% of the patients had orthostatic symptoms during HUTT, and 75% were asymptomatic [22]. Previous studies have shown that there was no significant co-occurrence of heart rate increment on head-up tilt table test and symptoms of orthostatic intolerance [26],[27]. The initial phase of active standing causes more drastic hemodynamic parameter change than the tilt-table test. Which distinctly explains patients' symptoms of orthostatic intolerance during the day to day life [28].

A total of 18 patients (45%) in our study had POTS with neurocardiogenic syncope on the head-up tilt table test during the nitroglycerin provocation phase. NCS may coexist with POTS in 38% of patients suffering from OI symptoms [29]. Satish Raj et al. reported that 30% of patients with POTS could have coexistent neurocardiogenic syncope [12]. In NCS, the patient's BP and HR maintained during head-up posture until a sharp drop in blood pressure, and often heart rate leads to presyncope and syncope [17]. An initial compensatory increment in sympathetic discharge that increases the heart rate and contractility of the heart may fatigue, or norepinephrine stores may become depleted. That is resulting in a state of relative sympathetic withdrawal, causing bradycardia and hypotension in this group of patients [18]. Physical manoeuvres such as leg crossing, muscle tensing, and squatting are useful in postponing and in some patients preventing NCS if used at the start of prodromal OI symptoms [30],[31].

Patient's with postural tachycardia syndrome have peripheral autonomic dysfunction. A subset of patients with POTS has low levels of standing plasma renin activity, aldosterone, and blood volume as compared to normovolemic patients [10]. There is also a surge of parasympathetic activity leading in marked cardioinhibition and vasodepression in POTS patients as described by Alshekhlee et al. [32]. This coexistence between POTS and NCS may be a spectrum of presentation and giving us a therapeutic window, by treating POTS we could curtail syncope and prevent grievous injury to patients because of syncope.

The management of our POTS patients included education and reassurance, non-pharmacological measures, and drugs, as no single therapy is effective. In our experience, most patients responded to counselling, regarding the essentiality of doing recumbent exercises, avoid long-standing and overheating, drink an excess of water, excess intake of salt, elevate the head end of the bed, elastic compression and beta-blocker therapy. In our study, all 15 patients, in those follow up of outcome was done, responded thoroughly for their symptoms with given management. An analysis of 56 patients done at Mayo clinic treated with non-pharmacological intervention and beta-blockers (54%), fludrocortisone (24%), found improvement in 70–80% of patients at the end of 1 year [33].

There were some limitations to the current study like an ambispective nature of the study with small number of patients. The treatment follow up was available for limited number patients.

## 6. Conclusion

POTS is a prevalent, under-diagnosed condition, and have a significant impact on the quality of life. Those patients come with generalized fatigue, heaviness overhead, and brain fog in everyday working should have been tested for postural orthostatic tachycardia. Orthostatic symptoms may not present during head-up tilt table test in a significant proportion of patients having POTS. So we have to be very vigilant while doing HUTT about variation in heart rate assessment that in the absence of symptoms, to diagnose POTS. NCS may coexist with POTS in a subgroup of patients suffering from orthostatic intolerance and diagnosis of this syndrome will help this group of highly symptomatic patients return to normal activities and gainful employment.
